# Modulatory Effect of Betulinic Acid on the Genotoxicity Induced by Different Mutagens in V79 Cells

**DOI:** 10.1155/2016/8942730

**Published:** 2016-04-19

**Authors:** Nathália Oliveira Acésio, Pollyanna Francielli de Oliveira, Daiane Fernanda Pereira Mastrocola, Ildercílio Mota de Souza Lima, Carla Carolina Munari, Vânia Luiza Ferreira Lucatti Sato, Andressa Aparecida Silva Souza, Lúzio Gabriel Bocalon Flauzino, Wilson Roberto Cunha, Denise Crispim Tavares

**Affiliations:** Universidade de Franca, Avenida Dr. Armando Salles de Oliveira 201, 14404-600 Franca, SP, Brazil

## Abstract

Betulinic acid (BA) is a pentacyclic triterpene that can be isolated from many medicinal plants around the world. The aim of this study was to evaluate the genotoxic potential of BA and its effect on the genotoxicity induced by different mutagens in V79 cells using the cytokinesis-block micronucleus assay. Different BA concentrations were combined with methyl methanesulfonate (MMS), doxorubicin (DXR), camptothecin (CPT), and etoposide (VP-16). The frequencies of micronuclei in cultures treated with different BA concentrations did not differ from those of the negative control. Treatment with BA and MMS resulted in lower micronucleus frequencies than those observed for cultures treated with MMS alone. On the other hand, a significant increase in micronucleus frequencies was observed in cultures treated with BA combined with DXR or VP-16 when compared to these mutagens alone. The results showed no effect of BA on CPT-induced genotoxicity. Therefore, BA was not genotoxic under the present experimental conditions and exerted a different influence on the genotoxicity induced by different mutagens. The modulatory effect of BA depends on the type of mutagen and concentrations used.

## 1. Introduction

Natural products have traditionally formed the backbone of modern drug discovery programs. The interest in this research area has markedly increased with improved understanding of the biology of carcinogenesis and the identification of potential molecular targets to disturb this process. Indeed, 41% of small-molecule anticancer drugs and 65% of antibacterial drugs discovered between the 1940s and 2010 were either natural products or semisynthetic derivatives of natural products [1].

Within this context, terrestrial plants, especially higher plants, have a long history of use for the treatment of human diseases [2]. One of the classes of natural products widely found in higher plants is triterpenoids, metabolites of isopentenyl pyrophosphate oligomers chemically related to squalene. Triterpenoids form a large group of compounds that contain 30 carbon atoms arranged in five rings to which several oxygen atoms are attached. The biological effects of triterpenoids include bactericidal, anti-inflammatory, antiviral, cytotoxic, and anticancer activity [3].

**Figure 1:**
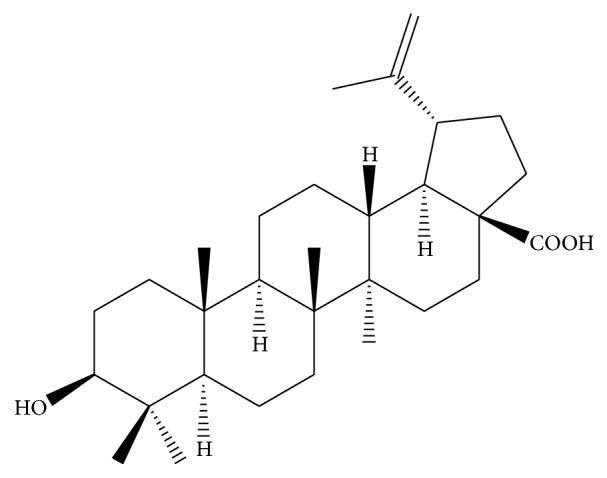
Chemical structure of the betulinic acid.

Betulinic acid (BA) (Figure 1) is a pentacyclic triterpenoid of plant origin occurring worldwide [4]. This triterpenoid has anti-inflammatory [5], antiviral [6], antibacterial, antiparasitic [7], and antitumor [8] activities. The antitumor activity of BA is mediated by the induction of apoptosis [3]. Betulinic acid has been shown to exert a synergistic cytotoxic effect on the growth and metastasis of melanoma cells (B16F10)* in vivo* when administered in combination with the anticancer drug vincristine [8]. Combined treatment with TRAIL (tumor necrosis factor- (TNF-) related apoptosis-inducing ligand) and BA cooperated to induce apoptosis in neuroblastoma cells, but not in normal human fibroblasts [9].

Since BA is a novel experimental antineoplastic agent and considering the need to elucidate the mechanism of action of new natural products, the aim of the present study was to evaluate the cytotoxic, genotoxic, and modulatory effect of BA on the genotoxicity induced by different mutagens in V79 cells. The results should contribute to a better understanding of the mechanism involved in the action of BA on DNA and its possible use as an adjuvant in cancer chemotherapy.

## 2. Materials and Methods

### 2.1. Plant Material and Isolation of Betulinic Acid

BA was obtained from the leaves of* Davilla elliptica* (St. Hill.; Dilleniaceae), collected in the Reserva de Jataí near the city of Luis Antônio, São Paulo State, Brazil. The plant was kindly identified by Dr. Milton Groppo, Departamento de Biologia, Faculdade de Filosofia, Ciências e Letras de Ribeirão Preto, Universidade of São Paulo (USP), where a voucher specimen has been deposited (voucher number SPFR 13702).

The plant material was dried in a stove under circulating air (40°C) and ground. The powdered material (770.0 g) was exhaustively extracted by maceration with* n*-hexane, ethyl acetate, and ethanol, in this order, at room temperature, yielding the crude extracts (5.8 g, 6.0 g, and 40.7 g, resp.).

An aliquot of the ethyl acetate extract was dissolved in methanol/water (1 : 1 v/v) and chromatographed over a preparative RP-HPLC Shimadzu Shim-pack ODS column (particle diameter 5 *μ*m, 250 × 20 mm) equipped with a precolumn of the same material, using methanol/water (88 : 12 v/v) as the mobile phase. After several injections at a flow rate of 9 mL min^−1^, betulinic acid (BA) was isolated and its chemical structure was confirmed by comparison of ^1^H- and ^13^C-NMR spectra [10]. The purity of the isolated compound was estimated to be higher than 95% by HPLC analysis and ^13^C-NMR spectroscopy.

### 2.2. Cells and Culture Conditions

Chinese hamster lung fibroblasts (V79 cells) were maintained as monolayers in plastic culture flasks (25 cm^2^) containing HAM-F10 (Sigma-Aldrich) and DMEM (Sigma-Aldrich) culture medium (1 : 1), supplemented with 10% fetal bovine serum (Nutricell), antibiotics (0.01 mg/mL streptomycin and 0.005 mg/mL penicillin; Sigma-Aldrich), and 2.38 mg/mL Hepes (Sigma-Aldrich), in a BOD incubator at 37°C. Under these conditions, the average cell cycle time was 12 h and the cells were used after the 4th passage.

### 2.3. DNA Damage-Inducing Agents

The following four mutagens with different mechanisms of action were used to study the modulatory effect of BA on DNA damage: (i) methyl methanesulfonate (MMS; Sigma-Aldrich) dissolved in phosphate-buffered saline (PBS) at a concentration of 400 *μ*M [11]. MMS is a direct-acting monofunctional alkylating agent that reacts with the DNA molecule by transferring methyl radicals [12]; (ii) doxorubicin (DXR; Eurofarma Laboratórios Ltda.) dissolved in sterile distilled water and used at a concentration of 0.3 *μ*M [13]. DXR, one of the most potent broad-spectrum antitumor anthracycline antibiotics, is a free radical generator and a potent inhibitor of topoisomerase II [14]; (iii) (S)-(+)-camptothecin (CPT; Sigma-Aldrich) dissolved in DMSO (Sigma-Aldrich) at a concentration of 123.4 *μ*M CPT acts by inhibiting topoisomerase I, an enzyme necessary for DNA replication [15]; (iv) etoposide (VP-16; Sigma-Aldrich) dissolved in DMSO at a concentration of 1.7 *μ*M. VP-16 is a potent anticancer agent that inhibits topoisomerase II [16].

### 2.4. Colony Formation Assay

The colony formation assay in V79 cells was used to assess the cytotoxicity of BA according to the protocol described by Franken et al. [17]. The cell cultures were treated with BA concentrations ranging from 0.3 to 44 *μ*M. Negative (no treatment), solvent (DMSO, 0.7 *μ*M), and positive (MMS, 1000 *μ*M) controls were included. The cultures were treated for 3 h and 300 cells were seeded per culture flask (three flasks per concentration). At the end of the growth period (10 days), the cells were fixed in methanol/acetic acid/distilled water (1 : 1 : 8) for 30 min and stained with 3% Giemsa for 30 min. The colonies formed were counted with a magnifying glass and the survival fraction (SF) was calculated for the different treatments using the following formula:

(1) $$\begin{array}{c} \text{SF}\left(\;\%\;\right)=\frac{A}{B}\times 100, \end{array}$$

where *A* is the number of colonies found in the different treatments and *B* is the number of colonies found in the negative control.

### 2.5. Cytokinesis-Block Micronucleus Assay

The concentrations of BA employed in the genotoxicity studies were chosen based on the results of the colony formation assay using cytotoxicity as a selection criterion. Thus, BA was evaluated at concentrations of 2.7, 5.5, 11, and 22 *μ*M. For assessment of the modulatory effect of BA, these concentrations were combined with the different mutagens described above. Negative (no treatment), solvent (DMSO, 0.3 *μ*M), and positive (DXR, MMS, CPT, and VP-16) controls were included. The protocol was performed in triplicate on three different days to ensure reproducibility.

The cytokinesis-block micronucleus assay was performed according to the protocol described by Fenech [18]. Approximately 500,000 cells were seeded in culture flasks and incubated in a BOD incubator for 25 h at 37°C. The cell cultures were then treated with the different concentrations of BA alone or combined with the different mutagens and incubated in a BOD incubator for 3 h at 37°C. After treatment, the cell cultures were washed with PBS and added to culture flasks containing 5 mL complete culture medium (10% fetal bovine serum) and cytochalasin B (6.3 *μ*M; Sigma-Aldrich). After 17 h of incubation, the cells were transferred to a microscope slide and stained with 3% Giemsa for 5 min.

The cells were analyzed by light microscopy using a 100x immersion objective. For each culture, 1,000 binucleated cells (3,000 cells per treatment) were analyzed and the number of cells containing 0, 1, 2, 3, or more micronuclei was counted. The cytotoxicity of the treatments was evaluated by calculating the nuclear division index (NDI). A total of 500 cells with well-preserved cytoplasm were evaluated per culture (1,500 cells per treatment) and the number of cells containing 1–4 nuclei was counted. The NDI was calculated according to Eastmond and Tucker [19] using the following formula:

(2) $$\begin{array}{c} \text{NDI: }\;\frac{\left[M1+2\left(M2\right)+3\left(M3\right)+4\left(M4\right)\right]}{N}, \end{array}$$

where *M*1 to *M*4 is the number of cells with 1, 2, 3, and 4 nuclei, respectively, and *N* is the total number of viable cells.

### 2.6. Statistical Analysis

The micronucleus test data were analyzed statistically by analysis of variance for completely randomized experiments, with calculation of *F* statistics and respective *P* values. In cases in which *P* < 0.05, treatment means were compared by the Tukey test and the minimum significant difference was calculated for *α* = 0.05.

## 3. Results 

**Figure 2:**
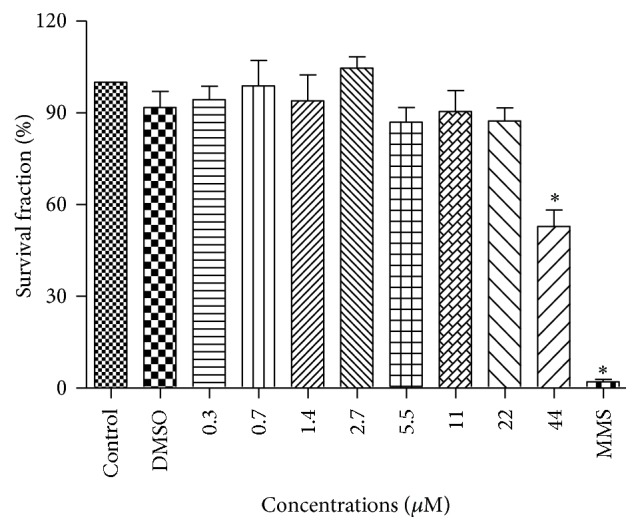
Survival fraction of V79 cells treated with different concentrations of betulinic acid. DMSO: dimethyl sulfoxide (0.3 *μ*M), MMS: methyl methanesulfonate (1000 *μ*M). ^*∗*^Significantly different from the control group (*P* < 0.05).

Figure 2 shows the results of the colony formation assay. No significant differences were observed between cultures treated with 0.3 to 22 *μ*M BA and the negative control, demonstrating the lack of a cytotoxic effect of BA on the cells. Therefore, concentrations of BA of 2.7, 5.5, 11, and 22 *μ*M were used in the subsequent experiments.

The frequencies of micronuclei in V79 cells treated with the different concentrations of BA are shown in Table 1. No significant differences in micronucleus frequency were observed when compared to the negative control group, indicating the absence of genotoxicity at all concentrations tested.

Table 2 shows the results of treatment with BA plus the different mutagens. Betulinic acid at a concentration of 5.5 *μ*M significantly reduced the frequency of micronuclei induced by MMS compared to cells treated with MMS alone. On the other hand, a significant increase in the frequency of micronuclei was observed for the combined treatment with BA (5.5 and 22 *μ*M) and DXR compared to treatment with DXR alone. Cultures treated with BA plus VP-16 also exhibited significantly higher micronucleus frequencies than those treated only with VP-16.

Regarding the combined treatment with BA and CPT, the frequencies of micronuclei of cell cultures treated with different concentrations of BA plus CPT did not differ significantly from those obtained for cells treated with CPT only.

Tables 1 and 2 also show the NDI values obtained for V79 cultures treated with the different concentrations of BA alone or combined with the different mutagens. No statistically significant difference was observed for any of the treatment groups when compared to the negative control, demonstrating the lack of cytotoxicity (*P* < 0.05).

## 4. Discussion

In the present study, BA was not genotoxic at any of the concentrations tested. A lack of genotoxicity has also been reported for another pentacyclic triterpene, that is, ursolic acid. The treatment of lymphocytes with ursolic acid caused no significant change in cell viability, thiobarbituric acid reactive substances, lipid hydroperoxides, percentage of DNA in the tail, or tail moment when compared to untreated lymphocytes [20].

With respect to the combined treatment with BA and different mutagens, we observed that BA was able to significantly reduce MMS-induced genotoxicity. MMS is a well-known DNA-damaging alkylating agent which forms DNA monoadducts and crosslinks that result in base substitution mutations [12]. Alkylating agents have also been reported to cause rapid depletion of glutathione S-transferase in mammalian cells, generating oxidative stress [21].

The loss of glutathione S-transferase has been postulated to compromise cellular antioxidant defenses, with the consequent accumulation of reactive oxygen species (ROS) generated as by-products of normal cellular function. The protection by antioxidants against damage caused by free radicals is of vital importance for the integrity of cells and macromolecules such as DNA. The generation of ROS may represent an alternative pathway in the genotoxicity induced by MMS.

The antioxidant potential of BA has been reported in the literature. Treatment of HepG2 cells with BA significantly reduced the ethanol-induced production of superoxide anion [22]. In another study, Szuster-Ciesielska et al. [23] reported significant inhibition of superoxide anion and hydrogen peroxide production by BA in liver stellate cells of mice treated with acetaldehyde, a reaction product of ethanol metabolism. In this respect, the antioxidant activity of BA may be responsible, at least in part, for the reduction in MMS-induced chromosome damage observed in the present study.

On the other hand, BA potentiated the DNA damage induced by DXR and VP-16. These mutagens are known inhibitors of topoisomerase II and are classified as topoisomerase poisons. The topoisomerase enzyme induces DNA double-strand breaks, relieving tension during DNA replication, recombination, and transcription. These breaks are transient and DNA repair is done by topoisomerase II itself [24]. Moreover, BA is a catalytic inhibitor of topoisomerase [25]. Catalytic inhibitors have been shown to reduce the clastogenicity of topoisomerase II poisons [26]. However, Ishida et al. [27] reported that catalytic topoisomerase II inhibitors exert both synergistic and antagonistic effects depending on treatment schedule and concentration. The potentiation of DNA damage observed in this study suggests that the mechanism of action of BA is directly related to the modulation of topoisomerase II activity, demonstrating a synergistic effect.

The present results also showed no influence of BA on the chromosome damage induced by CPT. This substance is a plant alkaloid originally isolated from the Chinese tree,* Camptotheca acuminata* Decne. CPT functions by binding to and stabilizing the covalent complex of the nicked topoisomerase I-DNA, which prevents DNA relegation and therefore causes irreversible DNA breaks during ongoing DNA and RNA synthesis [28]. According to Ganguly et al. [25], BA inhibits apoptosis by preventing the formation of cleavable topoisomerase I-DNA complexes mediated by CPT. Most substances that induce apoptosis in cells generate ROS which, in turn, cause oxidative damage to DNA. These oxidized bases are preferred sites for the formation of topoisomerase I-DNA cleavable complexes. Pretreatment with BA inhibited the formation of cleavable complexes mediated by CPT, interacting with the enzyme and preventing stabilization of the cleavable complex by CPT. However, when BA was added after treatment with CPT, it was unable to inhibit the formation of cleavable complexes, suggesting that BA exerts no effect on preformed topoisomerase I-DNA cleavable complexes [25]. This fact may explain the lack of influence of BA on CPT-induced genotoxicity observed in this study, since the cells were treated simultaneously with CPT and BA.

Betulinic acid showed no significant dose-response effect in the present assays. The assessment of dose-response effects is complicated by the fact that many compounds act simultaneously at different levels. The absence of a dose-response effect can be explained by several mechanisms, such as erratic absorption by the cell membrane and the consequent inconsistent bioavailability of the compound in the cell [29].

## 5. Conclusions

Under the present experimental conditions, BA was not genotoxic at any of the concentrations tested. This result contributes to the safety assessment of BA as a pharmaceutical intended for human use. However, this triterpenoid was effective in reducing the chromosome damage induced by the mutagen MMS. The antioxidant activity of BA may be responsible, at least in part, for the reduction in MMS-induced genotoxicity. On the other hand, BA potentiated the DNA damage induced by DXR and VP-16 but exerted no influence on the genotoxicity induced by CPT. Therefore, BA was able to modulate topoisomerase II activity but exerted no effect on preformed topoisomerase I-DNA cleavable complexes.

## Figures and Tables

**Table 1:** Frequency of micronuclei (MN) and nuclear division index (NDI) obtained for V79 cell cultures treated with betulinic acid.

Treatment (*μ*M)	MN frequency^a^	NDI^b^
Control	4.34 ± 0.58	1.69 ± 0.01
DMSO	3.67 ± 2.58	1.69 ± 0.03
2.7	5.34 ± 2.08	1.69 ± 0.01
5.5	5.67 ± 1.53	1.72 ± 0.01
11	4.67 ± 1.15	1.71 ± 0.02
22	7.67 ± 1.53	1.72 ± 0.02
MMS	42.34 ± 3.05^c^	1.72 ± 0.02

Results are reported as mean ± standard deviation. DMSO: dimethyl sulfoxide (0.3 *µ*M), MMS: methyl methanesulfonate (400 *µ*M). ^a^A total of 3,000 binucleated cells were analyzed per treatment group. ^b^A total of 1,500 cells were analyzed per treatment group. ^c^Significantly different from the negative control group (*P* < 0.05).

**Table 2:** Frequency of micronuclei (MN), nuclear division index (NDI), percent reduction, and increase in DNA damage obtained for V79 cultures treated with betulinic acid combined with different mutagens and the respective controls.

Treatment (*μ*M)	MN frequency^a^ (mean ± SD)	NDI^b^ (mean ± SD)
Control	4.34 ± 0.58	1.69 ± 0.01
DMSO	3.67 ± 2.58	1.69 ± 0.03

MMS	42.33 ± 3.05^c^	1.72 ± 0.02
DMSO + MMS	44.34 ± 7.63^c^	1.74 ± 0.01
2.7 + MMS	36.34 ± 1.52^c^	1.75 ± 0.07
5.5 + MMS	30.33 ± 1.52^c,d^	1.73 ± 0.05
11 + MMS	39.00 ± 2.64^c^	1.70 ± 0.02
22 + MMS	37.00 ± 4.58^c^	1.66 ± 0.09

DXR	24.00 ± 4.58^c^	1.68 ± 0.04
DMSO + DXR	30.67 ± 4.72^c^	1.73 ± 0.05
2.7 + DXR	47.33 ± 6.35^c^	1.75 ± 0.02
5.5 + DXR	116.67 ± 16.77^c,e^	1.70 ± 0.03
11 + DXR	47.34 ± 5.50^c^	1.70 ± 0.08
22 + DXR	66.67 ± 14.20^c,e^	1.69 ± 0.03

CPT	89.00 ± 6.24^c^	1.64 ± 0.02
DMSO + CPT	73.00 ± 1.00^c^	1.65 ± 0.01
2.7 + CPT	93.67 ± 3.05^c^	1.67 ± 0.01
5.5 + CPT	93.34 ± 3.05^c^	1.65 ± 0.02
11 + CPT	71.00 ± 6.24^c^	1.63 ± 0.02
22 + CPT	84.00 ± 16.37^c^	1.51 ± 0.01

VP-16	42.66 ± 1.52^c^	1.71 ± 0.04
DMSO + VP-16	38.33 ± 7.02^c^	1.78 ± 0.02
2.7 + VP-16	60.33 ± 1.52^c,f^	1.73 ± 0.01
5.5 + VP-16	62.66 ± 3.50^c,f^	1.69 ± 0.03
11 + VP-16	53.66 ± 4.72^c^	1.69 ± 0.03
22 + VP-16	61.33 ± 13.57^c,f^	1.73 ± 0.02

DMSO: dimethyl sulfoxide (0.3 *µ*M), MMS: methyl methanesulfonate (400 *µ*M), DXR: doxorubicin (0.3 *µ*M), CPT: (S)-(+)-camptothecin (123.4 *µ*M), VP-16: etoposide (1.7 *µ*M). ^a^A total of 3,000 binucleated cells were analyzed per treatment group. ^b^A total of 1,500 cells were analyzed per treatment group. ^c^Significantly different from the negative control group (*P* < 0.05). ^d^Significantly different from the MMS group. ^e^Significantly different from the DXR group. ^f^Significantly different from the VP-16 group.
